# Protective effect and mechanism of Shenkang injection on adenine-induced chronic renal failure in rats

**DOI:** 10.1590/acb370304

**Published:** 2022-06-01

**Authors:** Rongchang Chen, Lijiao Xu, Xu Zhang, Guibo Sun, Wenying Zeng, Xiaobo Sun

**Affiliations:** 1 MD. Peking Union Medical College and Chinese Academy of Medical Sciences - Institute of Medicinal Plant Development - Beijing, China.; 2 MM. Peking Union Medical College and Chinese Academy of Medical Sciences - Institute of Medicinal Plant Development - Beijing, China.; 3 MD. Xiyuan Hospital - Department of Comprehensive Medicine - Beijing, China.

**Keywords:** Kidney Failure, Chronic, Fibrosis, Rats

## Abstract

**Purpose:**

To investigate the protective effects of Shenkang injection (SKI) on adenine-induced chronic renal failure (CRF) in rat.

**Methods:**

Sprague Dawley rats were randomly divided into five groups: control, model, and SKI groups (5, 10, 20 mL/kg). Rats in model and SKI groups were treated with adenine i.g. at a dose of 150 mg/kg every day for 12 weeks to induce CRF. Twelve weeks later, SKI was administered to the rat i.p. for four weeks. The effects of SKI on kidney injury and fibrosis were detected.

**Results:**

SKI inhibited the elevation of the urine level of N-acetyl-b-D-glucosaminidase, kidney injury molecule-1, beta-2-microglobulin, urea protein in CRF rats. The serum levels of uric acid and serum creatinine increased and albumin decreased in the model group, which was prevented by SKI. SKI inhibited the release of inflammatory cytokines and increasing the activities of antioxidant enzymes in serum. SKI inhibited the expression of transforming growth factor-β1, vascular cell adhesion molecule 1, intercellular adhesion molecule 1, collagen I, collagen III, endothelin-1, laminin in kidney of CRF rats.

**Conclusions:**

SKI protected against adenine-induced kidney injury and fibrosis and exerted anti-inflammatory, and antioxidant effects in CRF rats.

## Introduction

The global incidence of chronic kidney disease (CKD) is about 10%, and nearly a third of patients with various primary or secondary CKD show some degree of renal failure^1^. Chronic renal failure (CRF) is defined as a progressive renal injury caused by CKD, which refers to a group of clinical syndromes with reduced glomerular filtration rate, abnormal renal metabolism and related clinical symptoms^2^. Nowadays, patients with end-stage CRF often need to rely on dialysis or kidney transplantation to maintain life, which has brought a heavy financial burden to family and society. Clinical medicine has paid more attention to seek effective medicines to slow, stop, or reverse the course of CRF.

Shenkang injection (SKI) is a traditional Chinese medicine consisting of four herbs: rhubarb (*Rheum palmatum* L.), Salvia miltiorrhiza (*Salvia miltiorrhiza* Bge.), safflower (*Carthamus tinctorius* L.), and Radix Astragali [*Astragalus membranaceus* (Fisch.) Bunge]. It has been widely used to treat CRF in China for decades. Many clinical observations show that SKI can ameliorate renal dysfunction effectively for CRF patients at stage III to stage IV^3^. The mechanism of renal protection by SKI may be related to suppressing kidney fibrosis and oxidative stress^4^. However, up to now, there are few preclinical research data of SKI and some important issues unresolved and unclearness of the functional mechanism, which limits its modernization and acceptance by Western medicine.

In the present study, we aimed to study the protective effect of SKI on adenine-induced CRF in rats and reveal its potential mechanism.

## Methods

All animal experiments were approved by the Animal Committee of Chinese Academy of Medical Sciences. A total of 70 male Sprague Dawley rats, weighing 250-300 g, were obtained from Beijing Vital River Laboratory Animal Technology Co., Ltd., with a certification number of SCXK (Beijing) 2017-0020.

### Chemicals and reagents

SKI was obtained from Xi’an Century Shengkang Pharmaceutical Industry Co., Ltd. (Xi’an, China). Adenine was purchased from Sigma-Aldrich (St. Louis, MO, United States of America). Primary antibodies against transforming growth factor-β1 (TGF-β1), vascular cell adhesion molecule 1 (VCAM-1), intercellular adhesion molecule 1 (ICAM-1), collagen I, collagen III, endothelin-1 (ET-1), and laminin (LN) were obtained from Abcam (Cambridge, United Kingdom). Kits of N-acetyl-b-D-glucosaminidase (NAG), kidney injury molecule-1 (KIM-1), beta-2-microglobulin (β2-MG), urea protein (UP), albumin (ALB), uric acid (UA), serum creatinine (Scr), catalase (CAT), superoxide dismutase (SOD), glutathione peroxidase (GSH-PX) and malondialdehyde (MDA) were purchased from BioSino Bio-Technology & Science Inc (Beijing, China). Enzyme-linked immunosorbent assay (ELISA) kits of interleukin-1β (IL-1β), interleukin-6 (IL-6), tumor necrosis factor-α (TNF-α), interleukin-4 (IL-4), interleukin-10 (IL-10), and C-reactive protein (CRP) were purchased from Sinoukbio (Beijing, China).

### CRF model establishment

Rats were housed in a controlled environment (21 ± 1°C, 55 ± 5% relative humidity, 12-h light/dark cycle) and were allowed free access to water and food. After one week of acclimation, 60 rats were given 150 mg/kg adenine suspension (freshly dissolved in 0.5% CMC-Na) i.g. for 12 weeks to induce CRF, and the remaining 10 rats, as the normal control group, were given an equal volume of 0.5% sodium carboxymethylcellulose (CMC-Na) i.g. Some rats died as the disease progressed.

### Animal grouping and drug treatment

**Figura 1:**
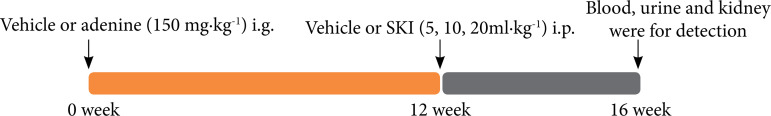
Protocol of *in-vivo* study.

As shown in Fig. 1, at the 12th week, rats with CRF were divided into four groups, model group and three SKI groups, 10 rats in each group. First, we divided the rats into three grades according to the BUN and Cre, and then divided rats in each grade into four groups according to the body weight, to ensure that the bias of body weight, BUN, or Cre is relatively small. The animals in three SKI groups were treated with SKI (5, 10, 20 mg/kg) i.p. for four weeks. The clinical equivalent dose is 10 mL/kg SKI. Control and model groups were given the same volume of normal saline.

### Measurements of renal injury factors in urine

After treatment, 24-h urine was collected by metabolic cage. UP concentration and NAG, KIM-1, β2-MG levels were determined by an automatic biochemical analyzer (Mairui BS-420, China).

### Detection of Scr, UA, ALB, CAT, MDA, SOD, and GSH-PX levels in serum

Rats were weighted, fasting (overnight) blood samples were collected from the abdominal aorta in heparinized tubes, allowed to clot for 30 min, and centrifuged at 3,000 rpm for 10 min at 4°C. The levels of Scr, UA, and ALB were detected by an automatic biochemical analyzer (Mairui BS-420, china). Serum concentrations of CAT, MDA, SOD, and GSH-PX were measured using assay kits according to the manufacturer’s protocol.

### ELISA for detection of IL-1β, IL-6, TNFα, CRP, IL-4, and IL-10 levels in serum

The concentrations of IL-1β, IL-6, TNFα, CRP, IL-4, IL-10 levels in serum were detected by rat-specific ELISA kit according to the manufacturer’s protocol. Enzyme labeling analyzer (Huaweidelang DR-200BS, China) was used to read the detection data.

### Hematoxylin and Eosin and Masson staining and immunohistochemical analyses

After blood sampling, the kidneys were dissected out and weighed. The ratio of kidney weight to body weight (defined as the kidney index) was calculated accordingly. The left kidney was fixed in 4% paraformaldehyde, dehydrated in graded ethanol, and embedded in paraffin max. The kidney apex was sectioned and stained with hematoxylin and eosin. Masson’s trichrome staining was used to examine extracellular matrix deposition. Structure was then examined under a light microscope (CKX41, 170, Olympus, Tokyo, Japan) by a pathologist blinded to the groups under study.

Immunohistochemical (ICH) staining of tissue sections was performed as described in a previous study^5^. Slides were deparaffinized and hydrated, and endogenous peroxidase was blocked by hydrogen dioxide. Sections were incubated with monoclonal antibody against ET-1, ICAM-1, VCAM-1, LN, Col I, Col III, and TGF-β1. The slides were washed in phosphate-buffered saline (PBS) and stained using DAB kit. Finally, the slides were restrained with hematoxylin, mounted, and observed under a light microscope.

Staining was carefully quantified in each slide by capturing ten randomly chosen fields in a blind manner by two experienced renal pathologists. Briefly, the ratio of positive staining area to the total area was calculated. These data were analyzed using Image-Pro Plus software (Media Cybernetics, Rockville, MD, United States of America).

### Statistical analysis

All data are presented as mean ± standard deviation. Statistical significance was determined using one-way analysis of variance followed by least significant difference (LSD) test for multiple comparisons, using Statistical Package for the Social Sciences (SPSS) version 17.0 statistical software (IBM, Armonk, NY, United States of America). The significance level was set at P < 0.05.

## Results

### Effects of SKI on body weight, renal weight, renal index, and urine volume (24 h) in CRF rats

As shown in Table 1, compared with the control group, the body weight and renal weight of rats in the model group decreased significantly. SKI (10 and 20 mL/kg) increased the body weight of CRF rats remarkably compared with the model group. However, SKI treatment had no significant effect on kidney weight. Compared with the control group, renal index and 24-h urine volume of rats in the model group increased significantly. SKI treatment decreased renal index in 20 mL/kgand reduced 24-h urine volume in 10 and 20 mL/kg significantly.

**Table 1:** Effects of Shenkang injection (SKI) on body weight, renal index, and 24-h urine volume in chronic renal failure rats*.

	Control (10 samples)	Model (10 samples)	SKI 5 mL·kg^-1^ (10 samples)	SKI 10 mL·kg^-1^ (10 samples)	SKI 20 mL·kg^-1^ (10 samples)
Body weight (g)	602.5 ± 56.99	442.1 ± 51.37**	478.2 ± 60.53	499.5 ± 44.53#	505.9 ± 61.08#
Renal weight (g)	1.71 ± 0.19	2.54 ± 0.48**	2.66 ± 0.55	2.55 ± 0.45	2.35 ± 0.30
Renal index	2.85 ± 0.31	5.86 ± 1.46**	5.59 ± 1.12	5.10 ± 0.79	4.71 ± 0.79#
Urine volume (mL)	20.25 ± 4.20	45.71 ± 8.53**	41.42 ± 13.23	35.42 ± 6.27##	33.22 ± 10.00##

* Data are means ± standard deviation;

** p < 0.01 *vs*. control group;

# p < 0.05;

## p < 0.01 *vs*. model group.

### Effects of SKI on the levels of NAG, KIM-1, β2-MG, and UP in urine of CRF rats

NAG, KIM-1, β2-MG, and UP are four important biomarkers of kidney injury. The significant increase of their levels in urine indicates kidney injury. As shown in Table 2, compared with the control group, the levels of NAG, KIM-1, β2-MG, and UP in rat urine of the model group elevated significantly, which was inhibited by SKI (10 and 20 mL/kg) significantly.

**Table 2:** Effects of Shenkang injection (SKI) on the levels of NAG, KIM-1, β2-MG,and UP in urine of chronic renal failure rats*.

	Control (10 samples)	Model (10 samples)	SKI 5 mL·kg^-1^ (10 samples)	SKI 10 mL·kg^-1^ (10 samples)	SKI 20 mL·kg^-1^ (10 samples)
NAG (μmol/L)	23.67 ± 4.71	51.52 ± 7.25**	43.82 ± 10.16	35.26 ± 7.34##	32.34 ± 8.19##
KIM-1 (ng/L)	14.15 ± 3.39	28.01 ± 2.75**	26.19 ± 2.94	22.92 ± 4.97#	18.71 ± 2.54##
β2-MG (ng/L)	43.53 ± 7.31	77.40 ± 4.23**	69.32 ± 13.17	65.25 ± 11.33##	55.91 ± 9.45##
UP (μmol/L)	5.66 ± 1.94	16.56 ± 2.51**	16.95 ± 4.25	12.60 ± 2.47##	10.08 ± 2.67##

* Data are means ± standard deviation;

** p < 0.01 *vs*. control group;

# p < 0.05;

## p < 0.01 *vs*. model group; NAG: N-acetyl-b-D-glucosaminidase; KIM-1: kidney injury molecule-1; β2-MG: beta-2-microglobulin; UP: urea protein.

### Effects of SKI on the levels of Scr, UA, and ALB in serum of CRF rats

As shown in Table 3, the serum levels of Scr and UA in the model group were higher than in the control group, and ALB was lower than in the control group. SKI treatment decreased the serum levels of Scr and UA and increased the level of ALB significantly.

**Table 3:** Effects of Shenkang injection (SKI) on the levels of Scr, UA, and ALB in serum of chronic renal failure rats*.

	Control (10 samples)	Model (10 samples)	SKI 5 mL·kg^-1^ (10 samples)	SKI 10 mL·kg^-1^ (10 samples)	SKI 20 mL·kg^-1^ (10 samples)
Scr (μmol/L)	34.17 ± 6.10	89.47 ± 32.48**	55.48 ± 20.54#	52.22 ± 13.28##	47.78 ± 10.55##
UA (μmol/L)	2.84 ± 1.14	25.39 ± 7.08**	26.56 ± 3.58	11.83 ± 4.39##	4.53 ± 1.37##
ALB (g/L)	11.37 ± 4.77	3.53 ± 1.05**	6.42 ± 3.06#	5.17 ± 1.24#	5.22 ± 1.76#

* Data are means ± standard deviation;

** p < 0.01 *vs*. control group;

# p < 0.05;

## p < 0.01 *vs*. model group; Scr: serum creatinine; UA: uric acid; ALB: albumin.

### Effects of SKI on the levels of CAT, SOD, GSH-PX, and MDA in serum of CRF rats

The level of MDA and the activities of SOD, GSH-PX, and CAT in serum were detected. As shown in Table 4, compared with the control group, the activities of SOD, GSH-PX, and CAT in the model group decreased significantly, and the level of MDA increased significantly. Compared with the model group, SKI decreased the level of MDA at 20 mL/kg significantly and increased the levels of SOD, GSH-PX, and CAT at 10 and 20 mL/kg significantly.

**Table 4:** Effects of Shenkang injection (SKI) on the levels of CAT, SOD, GSH-PX, and MDAin the serum of chronic renal failure rats*.

	Control (10 samples)	Model (10 samples)	SKI 5 mL·kg^-1^ (10 samples)	SKI 10 mL·kg^-1^ (10 samples)	SKI 20 mL·kg^-1^ (10 samples)
CAT (U/mL)	89.05 ± 10.06	54.56 ± 14.75**	59.59 ± 10.92	73.11 ± 21.75#	79.81 ± 4.95##
SOD (U/mL)	108.62 ± 17.62	56.11 ± 6.84**	64.86 ± 16.49	87.49 ± 7.28##	107.85 ± 7.98##
GSH-PX (U/mL)	817.36 ± 112.74	473.77 ± 62.55**	537.90 ± 96.38	687.39 ± 115.48##	736.47 ± 90.17##
MDA (nmol/mL)	3.27 ± 0.48	5.20 ± 0.60**	5.03 ± 0.66	4.94 ± 0.70	3.90 ± 0.36##

* Data are means ± standard deviation;

** p < 0.01 *vs*. control group;

# p < 0.05;

## p < 0.01 *vs*. model group; CAT: catalase; SOD: superoxide dismutase; GSH-PX: glutathione peroxidase; MDA: malondialdehyde.

### Effects of SKI on levels of CRP, IL-1β, IL-6, TNFα, IL-4, and IL-10 in serum of CRF rats

CRP is a nonspecific marker of systemic inflammation. The level of CRP in the model group increased significantly, which indicated a significant inflammatory response. However, SKI treatment reduced the level of CRP significantly. Compared with the control group, the level of pro-inflammatory factors, IL-1β, IL-6, and TNFα, decreased significantly, and the level of anti-inflammatory factors, IL-4 and IL-10, increased significantly in the model group. SKI alleviated inflammatory response by inhibiting IL-1β, IL-6, and TNFα and activating IL-4 and IL-10 (Table 5).

**Table 5:** Effects of Shenkang injection (SKI) on the levels of CRP, IL-1β, IL-6, TNF-α, IL-4,and IL-10 in serum of chronic renal failure rats*.

	Control	Model	SKI 5 mL·kg^-1^	SKI 10 mL·kg^-1^	SKI 20 mL·kg^-1^
CRP (mg/L)	2.60 ± 0.68	5.59 ± 1.19**	4.47 ± 0.81#	3.22 ± 1.63##	3.33 ± 0.57##
IL-1β (pg/mL)	16.98 ± 2.89	41.42 ± 6.64**	33.98 ± 7.88#	22.99 ± 11.84##	22.00 ± 2.80##
IL-6 (pg/mL)	80.05 ± 10.38	177.61 ± 18.70**	148.65 ± 18.88##	94.87 ± 9.02##	95.88 ± 6.20##
TNF-α (pg/mL)	36.33 ± 7.90	74.86 ± 6.15**	65.09 ± 17.57	30.71 ± 5.71##	47.00 ± 3.73##
IL-4 (pg/mL)	16.36 ± 2.22	8.64 ± 1.02**	9.59 ± 1.28	6.16 ± 0.77##	13.24 ± 1.50##
IL-10 (pg/mL)	26.18 ± 3.09	15.34 ± 2.32**	17.66 ± 2.38#	20.23 ± 5.57#	20.52 ± 1.85##

* Data are means ± standard deviation;

** p < 0.01 *vs*. control group

# p < 0.05;

## p < 0.01 *vs*. model group; CRP: C-reactive protein; IL-1β: interleukin-1β; IL-6: interleukin-6; TNF-α: tumor necrosis factor-α; IL-4: interleukin-4; IL-10: interleukin-10.

### Effects of SKI on pathological alterations, ET-1 expression, and fibrosis in the kidney of CRF rats

**Figura 2:**
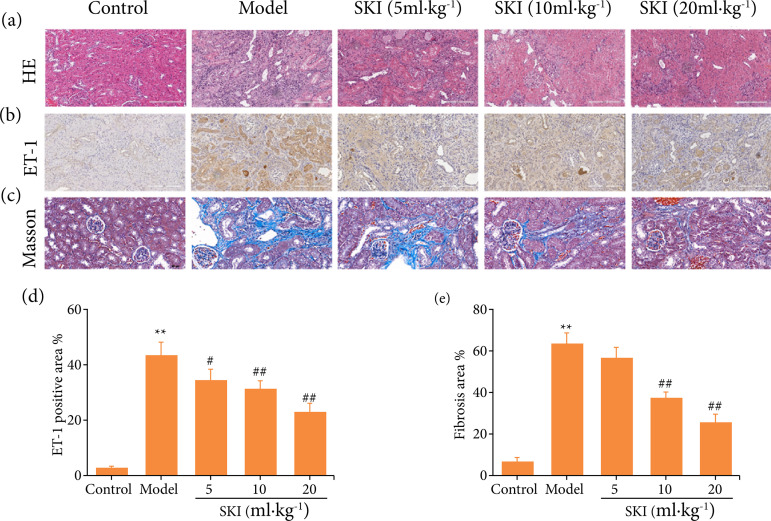
Effects of Shenkang injection (SKI) on kidney injury and fibrosis in chronic renal failure rats. **(a)** HE staining (scale bar = 200 μm); **(b)** ET-1 expression (scale bar = 200 μm); **(c)** Masson staining (scale bar = 50 μm) in kidney of chronic renal failure rats; **(d)** quantification of ET-1 expression; **(E)** Masson staining*.

HE: hematoxylin and eosin; ET-1: endothelin-1; *data are means ± standard deviation; **p < 0.01 *vs*. control group; ^#^p < 0.05; ^##^p < 0.01 *vs*. model group.

Compared with the control group, the kidneys in the model group showed glomerular deformation and vacuolar degeneration; the renal tubules around the glomerular were atrophic, dilated and there was brown matter deposition in the lumen, renal interstitial fibrosis and inflammatory cells infiltration. These pathological alterations were significantly attenuated with SKI treatment (Fig. 2a). ET-1 is a potent endothelium-derived vasoconstrictor peptide, and its high level is associated with increased risk of mortality in patients with chronic kidney failure^6^. Results from ICH showed that ET-1 was highly expressed in the kidney of the model group, which was inhibited by SKI treatment (Figs. 2b and 2d). Masson trichrome staining indicated that adenine induced a significant increase of collagen fibrils (blue area) compared with the control group. SKI treatment attenuated the collagen accumulation significantly (Figs. 2c and 2e).

### Effects of SKI on expression of VCAM-1 and ICAM-1 in the kidney of CRF rats

**Figura 3:**
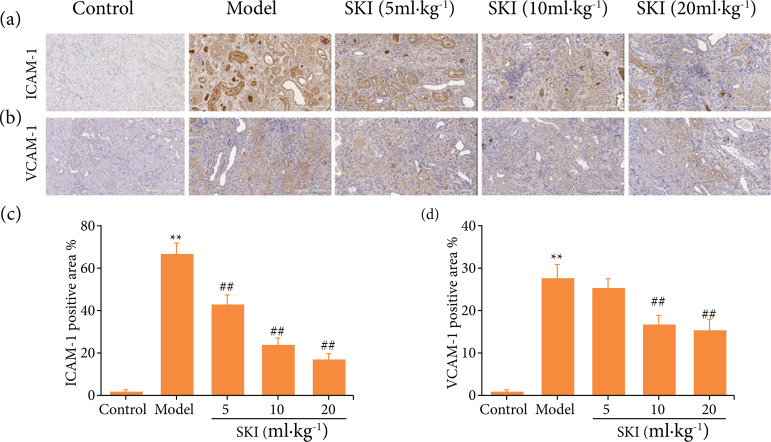
Immunohistochemical analysis of **(a, c)** ICAM-1 and **(b, d)**VCAM-1 in kidney of chronic renal failure rats (scale bar = 200 μm)*.

SKI: Shenkang injection; VCAM-1: vascular cell adhesion molecule 1; ICAM-1: intercellular adhesion molecule 1;*data are means ± standard deviation; **p < 0.01 *vs*. control group; ^#^p < 0.05; ^##^p < 0.01 *vs*. model group.

As shown in Fig. 3, compared with the control group, adenine exposure significantly increased the expression of VCAM-1 and ICAM-1, which were inhibited significantly by SKI treatment.

### Effects of SKI on expression of LN, collagen I, collagen III, and TGF-β1 in the kidney of CRF rats

**Figura 4:**
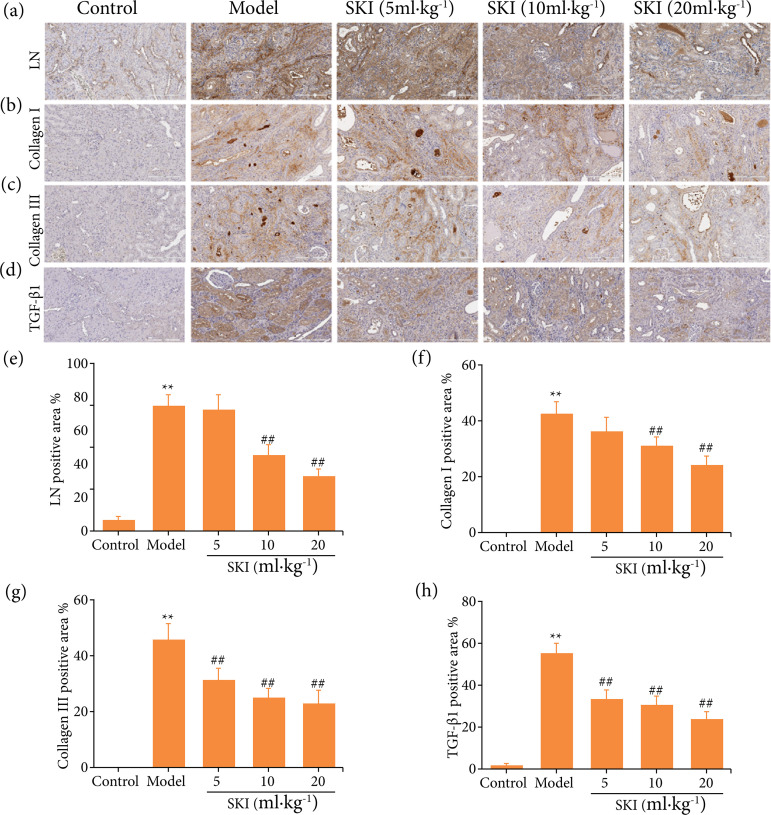
Immunohistochemical analysis of **(a, e)** LN, **(b, f)** collagen I, **(c, g)** collagen III, and **(d, h)** TGF-β1in kidney of chronic renal failure rats (scale bar = 200 μm)*.

LN is a kind of no collagen sugar, and its concentration reflects the degree of renal fibrosis^7^. As shown in Fig. 4, compared with the control group, adenine exposure significantly increased collagen I, collagen III, TGF-β1, and LN expression. SKI treatment significantly inhibited LN, collagen I, collagen III, and TGF-β1 expression.

LN: laminin; TGF-β1 transforming growth factor-β1; *data are means ± standard deviation; **p < 0.01 *vs*. control group; ^#^p < 0.05; ^##^p < 0.01 *vs*. model group.

## Discussion

In the study, we demonstrated that SKI treatment prevented the injury and fibrosis of kidney in CRF rats. First, we found that SKI administration increased body weight, but had no effect on kidney weight in CRF rats. The renal index can reflect the severity of CRF^8^. Previous study found that feeding adenine resulted in the expected CRF in mice by increasing the renal index^9^. SKI decreased renal index and urine volume (24 h) in CRF rats. We next detected the levels of four important biomarkers of kidney injury, NAG, KIM-1, β2-MG, and UP, in rat urine. The levels of NAG, KIM-1, β2-MG, and UP in the model group elevated markedly, which was inhibited by SKI (10 and 20 mL/kg) significantly. Besides, we tested the content of Scr, UA, and ALB in serum, because they can also reflect the extent of kidney damage well^10^. The increase of Scr and UA and the decrease of ALB in the serum of model group were prevented by SKI treatment. These results further confirmed the strong protective effect of SKI on renal failure and provided a theoretical basis for the clinical application of SKI.

Oxidative stress is considered the key pathway in the process of CRF development. Reactive oxygen species (ROS) family includes molecular oxygen and its derivatives^11^. The excessive production of ROS can induce renal dysfunction^12^. Therefore, decreasing the level of ROS is essential to reduce kidney damage^13^. SOD, GSH-Px, and CAT are considered the most representative natural antioxidant enzymes against oxidative stress *in vivo*, forming the first defense against ROS in organisms^14^. In our study, SKI increased the SOD, GSH-Px, and CAT activities and decreased the MDA content of adenine-induced kidney injury group, indicating that the protective effect of SKI may be related with its antioxidant activity.

Chronic inflammation has been regarded as one of the prominent features of CRF that is characterized by enhanced inflammatory responses^15^. Elevation of serum inflammatory markers is often observed in patients with CRF^16^. IL-10 is an anti-inflammatory factor, and its deficiency aggravates renal inflammation, fibrosis, and functional failure^17^. Our study indicated that SKI alleviated inflammatory reaction by inhibiting IL-1β, IL-6, and TNFα and activating IL-4 and IL-10. Metabolic acidosis in CRF stimulates the production of ET-1, and its chronic upregulation promotes inflammation and fibrosis^18^. Results from ICH showed that the highly expressed of ET-1 in the model group was reduced by SKI treatment.

VCAM-1 and ICAM-1 are two important cell adhesion molecules, which participate in the adhesion and migration of immune cells and play an important role in the pathological process of inflammation, tumor metastasis, and autoimmune diseases. Studies have shown that immune inflammation is the basis of various renal diseases, and leukocyte adhesion is the key link in the inflammatory response^19^^,^ 20. Therefore, the role of adhesion molecules in renal diseases has been paid increasing attention. Our study showed that the expression level of VCAM-1 and ICAM-1 increased in rats with chronic renal failure, which was inhibited by SKI treatment.

Fibrosis is an important pathological feature of various organs in many diseases. Changes of kidney function in CRF are often accompanied by fibrosis^21^. Some literature studies reported that CRF rats developed progressive proteinuria, glomerular mesangial matrix dilatation, glomerulosclerosis, mesangial dilation, and increased type I, type III collagen and fibronectin protein levels in the kidney^22^. Similarly, our results also showed that adenine exposure increased the expression of fibrosis proteins, including collagen I, collagen III, and LN in CRF rats, and renal tubular epithelial destruction, glomerular hypertrophy, mesangial expansion, and renal fibrosis were observed. SKI treatment attenuated the level of kidney fibrosis in CRF rats.

The occurrence and development of renal fibrosis are a complex and dynamic process including inflammatory cell infiltration, fibroblast activation and proliferation, tubular atrophy, and microvascular degeneration^23^. A lot of genes have been reported to be involved in this process. TGF-β1 plays a key role in initiating the occurrence and accelerating the progress of fibrosis^24^. We found that the expression level of TGF-β1 increased in adenine induced CRF rats and inhibited by SKI treatment.

## Conclusions

SKI protected against adenine-induced kidney injury and interstitial fibrosis and exerted anti-inflammatory, and antioxidant effects in CRF rats. The molecular mechanism may be related with the inhibition of TGF-β1 signaling pathway. Our results provide a basis for the clinical application of SKI. However, the mechanism of effect SKI on CRF needs further study.
